# Metagenomic next-generation sequencing as an unconventional approach to warn of tumor cells in a patients with non-mucinous pneumonic-type lung adenocarcinoma: Case report

**DOI:** 10.1097/MD.0000000000032448

**Published:** 2022-12-23

**Authors:** Yuexiang Shui, Huabin Wang

**Affiliations:** a Department of Respiratory and Critical Medicine, Lanxi People’s Hospital, Lanxi, Zhejiang; b Central Laboratory, Affiliated Jinhua Hospital, Zhejiang University School of Medicine, Jinhua city, Zhejiang.

**Keywords:** bronchoalveolar lavage fluid, human chromosomal instability, metagenomic next-generation sequencing, pneumonic-type lung cancer, screening

## Abstract

Pneumonic-type lung cancer (PTLC) is a special type of lung cancer with cough and expectoration as the main clinical symptoms and inflammatory signals as the main imaging manifestations. PTLC can be easily misdiagnosed as pneumonia, and the diagnosis and treatment are always delayed. Metagenomic next-generation sequencing (mNGS), as an emerging and effective method to identify occult pathogens, has been gradually adopted by clinicians.

**Patient concerns:**

A 58-year-old woman with recurrent cough and expectoration was admitted to hospital on January 12th, 2022. She reported that she was diagnosed with pneumonia half a month ago, after treatment with expectorant and antibiotics for 5 days, the symptoms were relieved. However, the symptoms worsened again 10 days after stopping the drugs. On the current presentation, she denied exposure to patients with infection of COVID-19, smoking history, night sweats, weight loss, rash, joint pain, fever, and shortness of breath.

**Diagnoses:**

The patient was diagnosed with non-mucinous pneumonic-type lung adenocarcinoma according to the clinical symptoms, changes of CT scans after treatment and cytopathology examinations.

**Interventions and outcomes:**

The patient was initially diagnosed with pulmonary infection according to computerized tomography (CT) scan. Expectorant and antibiotics used. However, the symptoms worsened again 10 days after stopping the drugs. On her return visit, the CT scan did not showed obvious consolidation absorption and was similar to the previous imaging findings. mNGS was performed to detect the occult pathogens. None pathogen was detected, however, 39 copy number variations were found in Human Chromosomal Instability Analysis of mNGS indicating the presence of tumor cells. The cytopathology findings confirmed the presence of lung adenocarcinoma (non-mucinous adenocarcinoma). She was treated with targeted antitumor drugs, and the CT scan after 20 days of targeted antitumor therapy showed obvious absorption of the lesions.

**Lessons:**

mNGS may have potential value to screen tumor cells in bronchoalveolar lavage fluid of patients with PTLC, especially in the patients whose samples in bronchioli cannot be collected using existing sampling tools.

## 1. Introduction

Pneumonic-type lung cancer (PTLC) is a special type of lung cancer characterized by ground glass shadows or consolidation in imaging; mucinous adenocarcinoma is the most common pathological type of PTLC.^[1,2]^ However, not all PTLC are mucinous adenocarcinomas, 2.9% of the patients with invasive non-mucinous adenocarcinoma also present with pneumonia in imaging.^[1]^ Patients with PTLC can be easily misdiagnosed as common pneumonia by clinicians,^[2]^ especially the radiologists without a great deal of experience in the township hospitals.

Metagenomic next-generation sequencing (mNGS) is rapidly transitioning from scientific research clinical practice,^[3,4]^ and it has become an emerging and effective approach to identifying occult pathogens from a variety of sample types.^[5,6]^ Most mNGS related studies have focused on the clinical values of mNGS for diagnosis of occult pathogens.^[7,8]^ We present here an unconventional use of mNGS: warning of cancer via human chromosomal instability analysis of bronchoalveolar lavage fluid in a patient with PTLC. To the best of our knowledge, this is the first report of warning of cancer in a patient with PTLC using mNGS.

## 2. Case report

**Figure 1.**
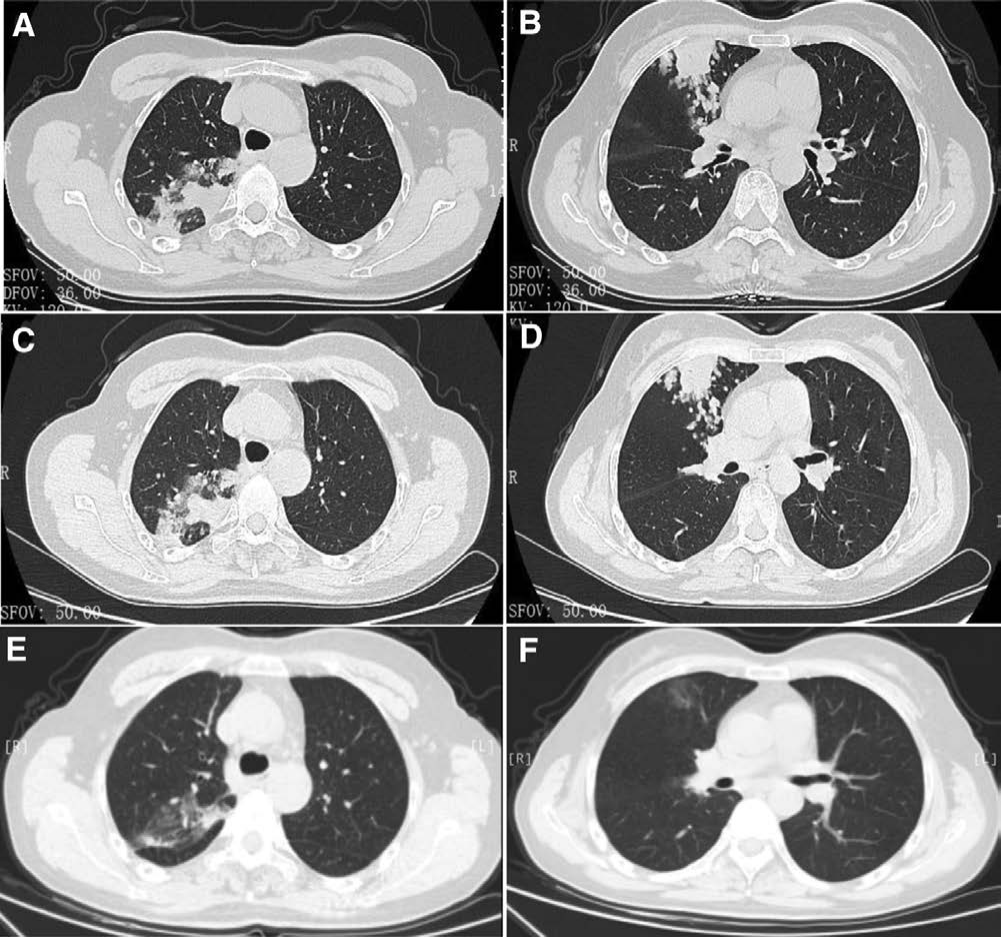
The chest CT scans of the patient. (A and B) The chest CT scan at initial diagnosis. (C and D) The chest CT scan after antibiotic therapy. (E and F) The chest CT scan after 20 days of targeted antitumor therapy. CT = computerized tomography.

On December 25th, 2021, a 58-year-old woman with cough and expectoration for more than 10 days visited respiratory outpatient department. Chest computerized tomography (CT) scan showed scattered nodular high-density shadows in both lungs, flaky high-density shadows in right upper and lower lobes, a ground glass-like high-density shadow (15 mm) in right upper lobe (Fig. 1A and B). The patient was diagnosed with pneumonia and admitted to hospital that day. On physical examination, the patient presented cough, expectoration, afebrile, normal weight. Vital signs were within the normal range, including a pulse rate of 83 beats/min and a respiratory rate of 20 times/min. The chest examination indicated coarse breath sounds on both lungs without obvious moist rales and rhonchi. The cardiac examination showed normal heart sounds without murmurs and expansion of cardiac boundary. Neurologic examination showed no abnormalities. She denied exposure to patients with infection of COVID-19, smoking history, night sweats, weight loss, rash, joint pain, fever, and shortness of breath. In general, the patient presented a good state of spirit.

The blood tests showed that carcinoma embryonic antigen, carbohydrate antigen 199, carbohydrate antigen 125, cytokeratin 19 fragment, neuron-specific enolase, and squamous cell carcinoma associated antigen were all within the normal ranges. Sputum acid-fast bacillus smear test showed negative. Treatment with expectorant and antibiotics relieved her symptoms and she was discharged 5 days later. She was requested to make a return visit in 1 month.

**Figure 2.**
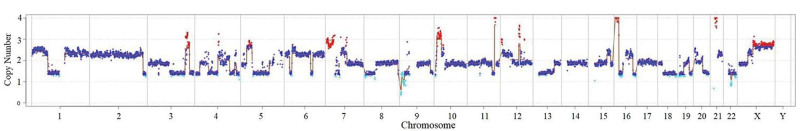
The result of human chromosomal instability analysis of bronchoalveolar lavage fluid using mNGS. Copy number > 2: duplication of chromosome; Copy number < 2: deletion of chromosome. mNGS = metagenomic next-generation sequencing.

**Figure 3.**
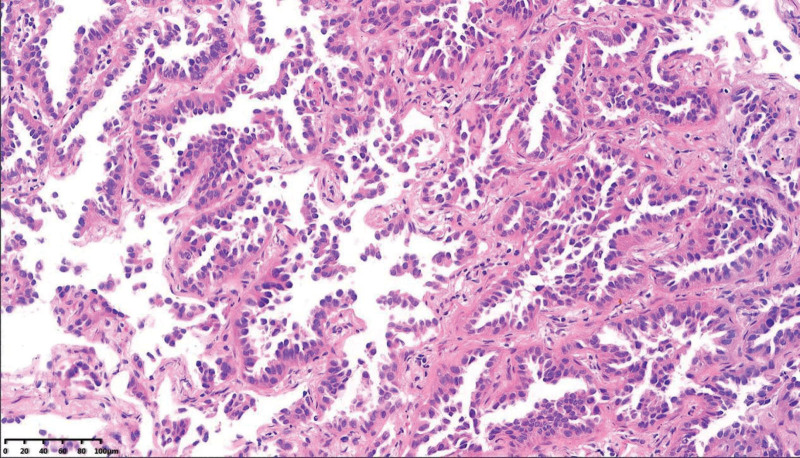
Lung adenocarcinoma was confirmed by HE staining of lung biopsy tissue, HE = hematoxylin-eeosin.

On the return visit (January 12th, 2022), the CT scan did not showed obvious consolidation absorption and was similar to the previous imaging findings (Fig. 1C and D). The patient was diagnosed with pulmonary infection (tuberculosis or other disease?) and admitted to hospital again. Endobronchial ultrasonography (EBUS) with guide–sheath was performed, and bronchoalveolar lavage fluid was detected to look for pathogens using mNGS. The mNGS results showed that none pathogen was detected, however, 39 copy number variations were found in the human chromosomal instability analysis of mNGS indicating the presence of tumor cells (Fig. 2 and Table 1). Four days later T4N0M0 lung adenocarcinoma (non-mucinous adenocarcinoma) was confirmed according to the pathologic findings and previous CT reports (Fig. 3).

**Table 1:** List of copy number variations of tumor cells of the patient.

Reads counts	G+C bases content	Copy number variations	Position and magnification of variations
17705748	0.397	7p22.3-p14.2(dup_36.9Mb)	chr7: 0-36900000_3.03_dup
		10p14-p12.1(dup_20.9Mb)	chr10: 6700000-27600000_3.10_dup
		21q11.1-q21.3(dup_13.5Mb)	chr21: 14300000-27800000_3.99_dup
		1p36.33-p31.1(dup[mos]_70.1Mb)	chr1: 700000-70800000_2.45_dup[mos]
		1p31.1-p11.2(del[mos]_50.6Mb)	chr1: 70900000-121500000_1.42_del[mos]
		1q21.1-q44(dup[mos]_103.85Mb)	chr1: 145400000-249250621_2.35_dup[mos]
		3q11.1-q25.33(del[mos]_66.9Mb)	chr3: 93500000-160400000_1.40_del[mos]
		3q26.33-q29(del[mos]_17.5Mb)	chr3: 180400000-197900000_1.42_del[mos]
		4p16.3-p15.32(del[mos]_17.1Mb)	chr4: 0-17100000_1.38_del[mos]
		4q13.3-q22.3(del[mos]_23.9Mb)	chr4: 72600000-96500000_1.41_del[mos]
		4q31.22-q32.3(del[mos]_21.8Mb)	chr4: 148100000-169900000_1.43_del[mos]
		4q34.2-q35.2(del[mos]_13.9Mb)	chr4: 177200000-191100000_1.37_del[mos]
		5p14.3-p12(dup[mos]_23.7Mb)	chr5: 21600000-45300000_2.65_dup[mos]
		5q11.1-q23.1(del[mos]_71.5Mb)	chr5: 49400000-120900000_1.46_del[mos]
		6p25.3-p22.3(dup[mos]_19.0Mb)	chr6: 100000-19100000_2.27_dup[mos]
		6p22.3-p22.1(del[mos]_10.1Mb)	chr6: 19100000-29200000_1.42_del[mos]
		6p22.1-p11.1(dup[mos]_29.6Mb)	chr6: 29200000-58800000_2.39_dup[mos]
		6q11.1-q21(dup[mos]_49.5Mb)	chr6: 61800000-111300000_2.28_dup[mos]
		6q22.31-q27(dup[mos]_49.9Mb)	chr6: 121100000-171000000_2.28_dup[mos]
		7p14.2-p11.1(del[mos]_21.2Mb)	chr7: 36900000-58100000_1.70_del[mos]
		7q11.21-q11.23(dup[mos]_12.7Mb)	chr7: 63300000-76000000_2.46_dup[mos]
		8p23.3-p11.1(del[mos]_43.7Mb)	chr8: 100000-43800000_1.37_del[mos]
		9q33.3-q34.3(del[mos]_11.1Mb)	chr9: 130000000-141100000_1.46_del[mos]
		11q22.3-q23.2(del[mos]_10.6Mb)	chr11: 103900000-114500000_1.41_del[mos]
		12p13.32-p11.1(dup[mos]_31.1Mb)	chr12: 3800000-34900000_2.30_dup[mos]
		13q11-q31.2(del[mos]_68.9Mb)	chr13: 19200000-88100000_1.43_del[mos]
		15q24.2-q26.1(dup[mos]_15.7Mb)	chr15: 76600000-92300000_2.36_dup[mos]
		16p13.11-p11.2(del[mos]_17.9Mb)	chr16: 16200000-34100000_1.37_del[mos]
		16q23.1-q24.3(del[mos]_12.1Mb)	chr16: 78100000-90200000_1.34_del[mos]
		18q11.1-q23(del[mos]_59.58Mb)	chr18: 18500000-78077248_1.38_del[mos]
		19p13.3-p12(del[mos]_16.0Mb)	chr19: 5200000-21200000_1.38_del[mos]
		19q13.32-q13.43(del[mos]_13.4Mb)	chr19: 45700000-59100000_1.38_del[mos]
		20q11.23-q13.33(del[mos]_27.8Mb)	chr20: 35200000-63000000_1.44_del[mos]
		21q21.3-q22.3(dup[mos]_20.3Mb)	chr21: 27800000-48100000_2.31_dup[mos]
		22q11.1-q12.1(del[mos]_11.4Mb)	chr22: 16100000-27500000_1.46_del[mos]
		22q12.2-q13.33(del[mos]_19.8Mb)	chr22: 31400000-51200000_1.37_del[mos]
		4q12-q13.2(del[mos]_14.2Mb)	chr4: 55900000-70100000_1.47_del[mos]
		Xp11.4-p11.1(dup[mos]_17.4Mb)	chrX: 41200000-58600000_2.34_dup[mos]
		Xq11.2-q28(dup[mos]_91.7Mb)	chrX: 63300000-155000000_2.69_dup[mos]

Due to the EGFR exon 19 deletion-insertion mutation (p.L747_A750delinsP), she was treated with targeted antitumor drugs and without surgery and antibiotics. The CT scan after 20 days of targeted antitumor therapy showed obvious absorption of the lesions (Fig. 1E and F), and confirmed that the inflammatory signals showed in imaging were actually the cancer lesions, not the inflammatory lesions. Finally, non-mucinous pneumonic-type lung adenocarcinoma was diagnosed according to the clinical symptoms, changes of CT scans and pathology examinations.

## 3. Discussion

With the progress of technology, EBUS combined with rapid on-site evaluation of cytology allow to make a decision rapidly during lung biopsy operation.^[9]^ However, the diagnostic yield in EBUS of patients with cancer is significantly associated with the tumor size.^[10]^ Due to the technical and tracheal structural burdens, the sampling tools cannot get into the smaller bronchioles for sampling, and the smaller lesion involve less bronchioles, the diagnostic yield is affected.^[9]^ Therefore, a more sensitivity and convenient method is needed for this situation.

Human chromosomal instability has been considered as an important cancer marker by many scientific researches. It can be applied to identify cancer in a form of noninvasive detection,^[11]^ however, it has rarely been used in clinical practice.^[4]^ mNGS is a technique method of high throughput sequencing of nucleic acid with fast turn-around time, high sensitivity and specificity, and it is usually used in clinical identification of pathogens in patients with infection diseases. In the present case, pulmonary infection was suspected initially, mNGS was performed to identify the pathogens; then the human chromosomal instability of exfoliated tumor cells in alveolar lavage fluid was sensitively detected by mNGS, indicating the potential value of mNGS for warning of tumor cells in patients with PTLC.

To the best of our knowledge, this is the first report of mNGS as an unconventional approach to warn of cancer via the human chromosomal instability analysis of bronchoalveolar lavage fluid in a patient with non-mucinous pneumonic-type adenocarcinoma. We believe that mNGS an unconventional approach has the potential to further improve diagnosis of lung cancer in patients with PTLC for 2 reasons. First, it is of high sensitivity and high throughput for nucleic-acid sequencing. Second, mNGS could warn of cancer by detecting the chromosomal instability of exfoliated tumor cells of alveolar lavage fluid from patients whose samples in bronchioli cannot be collected using existing sampling tools.

## Acknowledgments

The authors appreciate the expertise and contributions of Lixia Wang, Jiadong Yan.

## Author contributions

**Conceptualization:** Huabin Wang.

**Data curation:** Yuexiang Shui, Huabin Wang.

**Funding acquisition:** Yuexiang Shui.

**Visualization:** Huabin Wang.

**Writing – original draft:** Yuexiang Shui, Huabin Wang.
